# Adaptation of the Suicide Attempt Resilience Scale (SRSA-18, Spanish version) for adolescents

**DOI:** 10.1192/bjo.2022.601

**Published:** 2022-11-03

**Authors:** David Sánchez-Teruel, María Auxiliadora Robles-Bello, Aziz Sarhani-Robles, Mariam Sarhani-Robles

**Affiliations:** Department of Personality, Assessment and Psychological Treatment, University of Granada, Granada, Spain; Psychology Department, University of Jaen, Jaen, Spain; Faculty of Medicine, University of Granada, Granada, Spain; Faculty of Medicine, Autonomous University of Barcelona, Barcelona, Spain

**Keywords:** Resilience, assessment, SRSA-18, adolescents, protective factors

## Abstract

**Background:**

The assessment of resilience as an outcome in adolescents remains a challenge, with few instruments available. Some studies have focused on risk factors, but few have focused on protective factors as a formula for measuring resilient outcomes.

**Aims:**

To adapt a new Suicide Attempt Resilience Scale (SRSA-18) for use with adolescents, analysing its structural validity, the gender and age invariance of the measure, and divergent and convergent validity, together with its reliability.

**Method:**

The psychometric properties of the scale were assessed in 628 participants aged between 13 and 18 years, of whom 342 (54.5%) were girls.

**Results:**

After a process of adaptation for adolescents, exploratory and confirmatory factor analysis yielded a three-dimensional structure with adequate goodness-of-fit indices, invariance of the measure according to gender and age, adequate levels of reliability (ω = 0.91), high convergent validity with the 14-Item Resilience Scale and high divergent validity with the suicidal act/planning subdimension of the Adolescent Suicidal Behavior Assessment Scale.

**Conclusions:**

There is a need to create and adapt instruments to measure resilience in some populations with high psychosocial vulnerability as a key aspect for measuring the impact of prevention and mental health promotion programmes in adolescents.

Recent data indicate that suicide is the world's fourth leading cause of death in young people aged 15–19.^1^ These figures do not include suicide attempts, which appear to be up to 40 times more frequent than completed suicide.^2^ In addition, there are specific risk behaviours in children and adolescents, such as unintentional self-harm, that may desensitise them to more lethal future behaviours.^3^ Along these lines, suicide is less frequent in early childhood, but considerably more common in adolescence for both boys and girls.^4^

In Spain, the latest available data show that 77 children and adolescents (56 boys and 21 girls) aged 10–19 died by suicide in 2020.^5^ The difficulty in measuring the level of risk in adolescents in the general population highlights the ineffectiveness of some actions for early detection of self-harming behaviours in this group.^6^ Adolescence has specific characteristics related to the impact of specific adverse life events (family conflicts, marital break-ups, academic failure, peer relationship problems and bullying) that precede suicide attempts.^3,7^ This might suggest that the risk (and protective) factors related to suicide attempts and suicide at this stage of life could be different from those for adults.

In this regard, resilience is particularly important. It is a process in which the interaction of protective factors with risk factors after an adverse situation can produce appropriate personal growth and a final optimally adaptive outcome.^8^ Understanding of this aspect of being human originally emerged from the study of children and adolescents exposed to high-risk situations (e.g. maltreatment and sexual abuse) who did not develop psychopathological disorders, but managed to develop adequate levels of adaptation and a more constructive view of the adverse situations experienced.^9,10^ The concept of resilience is an attempt to understand and explain what aspects (protective factors) minimise psychopathological risk and promote positive development in people despite adversity.^8^ In fact, most studies on suicidal behaviour in adolescents have focused exclusively on risk variables.^11^ Some adolescents die on their first suicide attempt, others make more harmful subsequent attempts and others increase their level of resilience, enhancing the interaction between protective factors (internal, external or both) that minimise or reduce the psychosocial impact of risk factors, leading to a more resilient outcome.^8^ These differences in adolescents indicate an urgent need to develop instruments for assessing suicide attempt resilience in adolescents that focus exclusively on protective factors. Hence, the aim of this study was to adapt the Scale of Resilience to Suicide Attempts (SRSA-18) for use in adolescents and to provide data to verify its structure (exploratory and confirmatory factor analysis), reliability, and convergent and divergent validity, and to assess its invariance by gender and age.

## Method

### Participants

The initial sample comprised 801 participants; 70 were part of the pilot samples (see Procedure) and 103 were removed because they did not meet some of the inclusion criteria, which were: (a) being between 13 and 18 years old; (b) previous ‘self-injury’, ‘self-harming behaviour’ or ‘suicide attempt’; (c) completing all the questionnaires in their Spanish version; (d) being of Spanish nationality or living in Spain for more than 12 months; (e) reading the research information sheet and accepting and signing the informed consent (participants and parents or guardians); (f) parents or guardians expressly agreeing to their child's participation in the research; and (g) providing a parent's or guardian's email address. The final sample for psychometric analyses was made up of 628 participants aged between 13 and 18 years (mean age 15.11 years; s.d. = 4.2), 342 (54.5%) of whom were girls. The total sample was split into two for factor analysis:^12^ *n*_1_ = 298 participants, aged 13–18 years (mean age 14.97 years; s.d. = 5.33), 168 (56.4%) girls; and *n*_2_ = 330 participants, aged 13–18 (mean age 15.14 years; s.d. = 8.6), 174 (52.7%) girls. No statistically significant differences were found between the two subsamples (Table 1).

**Table 1:** Sociodemographic data of both samples

	Total sample (*n* = 628), *n* (%)	First subsample (*n*_1_ = 298), *n* (%)	Second subsample *n*_2_ = 330), *n* (%)	Student's *t*	Effect size, φ
Who do you currently live with?				3.11^n.s.^	0.63
Alone	11 (1.8)	5 (1.7)	6 (1.8)		
Partners or friends	118 (18.8)	52 (17.4)	66 (20.0)		
Alone with parents	196 (31.2)	92 (30.9)	104 (31.5)		
Parents and siblings	299 (47.6)	148 (49.7)	151 (45.8)		
Others	4 (0.6)	1 (0.3)	3 (0.9)		
Current education				1.16^n.s.^	0.73
None	21 (3.3)	8 (2.7)	13 (3.9)		
Primary	239 (38.0)	109 (36.6)	130 (39.4)		
Compulsory secondary	282 (45.0)	139 (46.6)	143 (43.3)		
Other	86 (13.7)	42 (14.1)	44 (13.4)		
Negative situation in the past 3 months				2.04^n.s.^	0.46
None	12 (1.9)	5 (1.7)	7 (2.1)		
Family problems	184 (29.3)	84 (28.2)	100 (30.3)		
Problems with friends	197 (31.4)	98 (32.9)	99 (30.0)		
Problems at school or high school	209 (33.3)	99 (33.2)	110 (33.3)		
Other	26 (4.1)	12 (4.0)	14 (4.3)		
What is the most important thing in your life?				1.01^n.s.^	0.84
Nothing	32 (5.1)	14 (4.7)	18 (5.5)		
Me	131 (20.9)	61 (20.5)	70 (21.2)		
My family	211 (33.6)	101 (33.9)	110 (33.3)		
My friends	227 (36.1)	110 (36.9)	117 (35.5)		
My classmates	12 (1.9)	5 (1.7)	7 (2.1)		
Other	15 (2.4)	7 (2.3)	8 (2.4)		
Type of suicidal act				1.55^n.s.^	0.76
Self-injury	129 (20.5)	61 (20.4)	68 (20.6)		
Self-harming behaviour	16 (2.6)	5 (1.7)	11 (3.3)		
Suicide attempt	483 (76.9)	232 (77.9)	251 (76.1)		
Incidents of self-injury, self-harming behaviour and/or suicide attempt, *n*				1.22^n.s.^	0.93
0	508 (80.9)	252 (84.6)	256 (77.6)		
1	106 (16.9)	41 (13.7)	65 (19.7)		
2 or more	14 (2.2)	5 (1.7)	9 (2.7)		
Total	628 (100)	298 (100)	330 (100)		

*P < 0.05; **P < 0.01; n.s. = not significant.

### Instruments

#### Sociodemographic data sheet

An *ad hoc* data sheet was prepared to record participants’ gender and age together with the data indicated in Table 1.

#### 14-Item Resilience Scale (RS-14) by Sánchez-Teruel & Robles-Bello^13^

This scale measures the degree of individual resilience allowing adaptation to adverse situations. The scale correlates negatively with depression and anxiety. Cultural and psychometric adaptation in young Spaniards demonstrates adequate internal consistency (α = 0.79), but presents a unifactorial structure.^13^ Reliability via Cronbach's alpha in the sample of this study was 0.75.

#### Adolescent Suicidal Behavior Assessment Scale (SENTIA) by Díez-Gómez et al^14^

This test measures suicidal behaviour based on risk factors in Spanish adolescents using 16 dichotomous (yes/no) statements. It has three subdimensions: suicidal act/planning, suicidal communication and suicidal ideation. The scale correlates positively with suicidal ideation, depression, emotional and behavioural problems and attenuated psychotic experiences and is not gender invariant. Overall internal consistency was ω = 0.91 (for acting/planning ω = 0.94; for communication ω = 0.84; and for ideation ω = 0.92).^14^ In this study only the suicidal act/planning subdimension with seven items was used, which gave coefficients of α = 0.89 and ω = 0.92.

#### Scale of Resilience to Suicide Attempts (SRSA-18) by Sánchez-Teruel et al^15^

This scale was constructed and validated in Spanish adults with previous suicide attempts and predicts future suicide reattempts^16^ using protective factors that enhance resilience. It consists of 18 items that are divided into three subdimensions: internal protection, emotional stability and external protection, with Likert-type responses from 0 (never) 4 (always). It correlates with other scales of resilience to stressful situations (Connor–Davidson Resilience Scale,^17^ CD-RISC: *r* = 0.79; *P* < 0.01; 14-Item Resilience Scale,^13^ RS-14, *r* = 0.76; *P* < 0.01) and suicidal ideation (Suicide Resilience Inventory-25,^18^ SRI-25, *r* = 0.91; *P* < 0.01) and exhibits a high level of internal consistency (α = 0.88; ω = 0.89).

### Procedure

In general, the initial implementation of the SRSA-18 in the three pilot samples was carried out face to face in the classrooms of the different schools and high schools that participated in this study, with the help of teachers and psychologists from these schools. Participants in the pilot samples were not part of the sample for further analysis. After this preliminary process, the rest of the participants completed all the measures online, as will be explained below. Data collection in all samples was anonymous. This requirement was strongly emphasised by both the parents and the young people themselves. The authors assert that all procedures contributing to this work comply with the Helsinki Declaration of 1975, as revised in 2008. All procedures involving human participants were approved by University of Jaen (approval ABR.20/4.PRY). This report includes the informed consent used.

The entire process of adapting and validating the SRSA-18 items for adolescents and young people followed several consecutive phases, as outlined in the following sections.^19,20^

### Administration to the first pilot sample (*n* = 11) and second pilot sample (*n* = 43)

First, to test comprehension of the SRSA-18,^21^ it was administered to 11 adolescents (first pilot sample) (aged 12–18 years; mean age 13.5 years; s.d. = 4.16; 7–63.6% girls), who exhibited difficulties in understanding (less than 3 points out of 5, where 0–5 is no–full understanding) original scale items 6 ‘Emotions don't overwhelm me’ and 16 ‘I control my impulses, even if I am pressured’. Using the participants’ suggestions these items were modified as: 6 ‘My actions are guided by my head, not my heart’ and 16 ‘I control my impulses’. Subsequently, the SRSA-18 was re-run on another subsample of 43 adolescents (second pilot sample) (aged 13–18 years; mean age 16.4 years; s.d. = 3.28; 22–51.2% girls), who exhibited difficulties in understanding 8 (1, 3, 11, 12, 13, 14, 15, and 17) of the 18 items of the original scale. These items were modified using the adolescents’ own suggestions together with a Spanish-speaking clinician, who re-evaluated whether the new wording was correct (Appendix 1).

### Expert panel, administration to third pilot sample (*n* = 16) and final version

Next, four experts (psychologists) in child and youth resilience determined whether or not all the new items corresponded to the psychological construct, which would help to ensure content validity.^22^ This led to only minor grammatical modifications to some of the items. Finally, the resulting scale was applied to a third pilot sample of 16 adolescents (10–62.5% girls) aged 13–18 years old (mean age 15.9 years; s.d. = 1.36). In this third pilot sample, discrimination and comprehension analyses of the items were performed and the time taken to complete the scale was assessed. The overall score was between 9 and 59 points (mean 42.7; s.d. = 6.31) with univariate normality. All items were easily understandable (above 0.60 on the difficulty index)^23^ and the corrected item-total correlation index (discrimination) was above 0.50.^24^ The average time to complete the scale was 16 min. The final version of this scale for adolescents is presented in Appendix 2.

### Psychometric properties of the SRSA-18 in adolescents and young people

First, approval for the study was sought from the University of Jaen's research ethics committee (ABR.20/4.PRY), in accordance with the Universal Declaration of Ethical Principles for Psychologists^25^ and the principles of the Declaration of Helsinki. Second, the quantitative sample (*n* = 628) was recruited online through a Google form link disseminated in participating schools and colleges by email. This study offered specific email feedback on the instruments applied to those parents or guardians who requested it.

### Data analysis

Item, internal consistency, convergent validity and discriminant validity analyses were conducted on the full sample (*n* = 628). The online administration of the tests meant that if information was not present, the test would not continue, so there were no missing data. An exploratory factor analysis (EFA) was performed on the first subsample (*n*_1_ = 298). The SRSA-18 presents a three-dimensional structure in a clinical sample of adults,^15^ which might suggest that EFA could be skipped. However, the use of a scale in a sample of a different age should be explored, as this aspect is key to construct validity.^26^ The structure of an assessment measure modulates the calculation of scores, so inaccurate or incorrect estimation may affect scores, applicability or the resulting clinical decisions.^27^ Hence, the initial exploration of dimensionality is a central feature of psychological research and a priority prior to any subsequent validation efforts. The EFA was performed using FACTOR 10.3 for Windows, which offers a semi-confirmatory method,^28^ through unweighted least squares with parallel analysis for factor extraction^29^ and the prominent method as rotation. Items that had a factor loading of less than 0.30 or that were complex (with cross loadings on several factors) were eliminated. A confirmatory factor analysis (CFA) was then performed on another subsample (*n*_2_ = 330) using the generalised least squares method. The fit indices used and satisfactory fit criteria were: χ^2^/d.f., root mean square error of approximation (RMSEA) close to 0.06 and goodness-of-fit index (GFI) close to 0.90, standardised root mean square residual <0.08, and comparative fit index (CFI) and the Tucker–Lewis index (TLI) ≥0.95.^30,31^ We examined whether there were differences in measurement invariance using multigroup CFA using AMOS (see below), specifying two nested models for gender and three models for age. The Satorra–Bentler scaled χ^2^ and its *P*-values, together with the RMSEA (95% CI) and CFI, were used for measurement invariance as an index of incremental fit.^32^ Configurational invariance (reference model) was used to test whether the groups associated the same subsets of items with the same constructs and with factor means set to zero. Metric invariance was to check whether the factor loadings between each item and its factor were the same across gender and age groups. Scalar invariance for all items was to measure whether the differences between the groups indicated by the items were the same.^33^ There is measurement invariance when *P* > 0.05 for Δχ^2^, the RMSEA is ≤0.05 and the ΔCFI value of the models compared is <0.01.^34^ Finally, reliability was assessed using McDonald's omega coefficient, convergent validity was assessed using the RS-14, and divergent validity was assessed with the subdimension of SENTIA. All analyses were performed with SPSS 26 AMOS for Windows^35^ and the minimum significance level was *P* < 0.05.

## Results

### Descriptive item analysis

Univariate normality was noted in the total sample (Table 2). Item-total correlations were adequate (*r*_i.t._ > 0.50), the difficulty index indicated no comprehension problems (*d*_i_ = 0.41–0.79) and item deletion (*α_−i_*) did not improve the level of internal consistency.

**Table 2:** Descriptive item analysis of the SRSA-18 for adolescents

Item	Mean (s.d.)	Kolmogorov–Smirnov test	Asymmetry,	Kurtosis,	*r*_i.t._	*d*_i_	*α_−i_*
			s.e. (0.16)	k.e. (0.31)			
1	1.27 (0.12)	0.09^n.s.^	0.21	0.43	0.61	0.49	0.53
2	3.18 (2.02)	0.53^n.s.^	0.34	0.87	0.73	0.41	0.69
3	0.12 (0.44)	0.66^1^	0.23	1.03	0.65	0.58	0.61
4	1.29 (0.43)	0.82^n.s.^	0.27	0.29	0.82	0.79	0.84
5	1.65 (0.13)	0.99^n.s.^	0.82	1.03	0.74	0.63	0.82
6	0.11 (0.27)	0.84^n.s.^	0.26	0.38	0.83	0.42	0.64
7	3.17 (0.11)	0.39^1^	0.65	0.93	0.66	0.58	0.87
8	0.19 (0.74)	0.63^n.s.^	0.32	0.48	0.69	0.76	0.84
9	3.21 (0.27)	0.15^n.s.^	0.43	0.71	0.59	0.48	0.81
10	0.14 (0.07)	0.74^n.s.^	0.61	0.93	0.72	0.56	0.73
11	2.11 (0.29)	0.38^n.s.^	0.64	0.68	0.81	0.65	0.83
12	1.21 (0.27)	0.73^n.s.^	0.55	0.73	0.88	0.53	0.74
13	0.31 (0.27)	0.19^n.s.^	0.14	0.93	0.82	0.42	0.69
14	2.19 (0.45)	0.39^n.s.^	0.77	0.82	0.78	0.75	0.61
15	1.11 (0.74)	0.72^n.s.^	1.29	1.12	0.91	0.49	0.78
16	2.31 (0.36)	0.69^n.s.^	0.01	0.34	0.63	0.56	0.69
17	3.22 (0.43)	0.82^n.s.^	0.43	0.11	0.83	0.44	0.83
18	2.71 (0.29)	0.19^n.s.^	1.01	1.26	0.78	0.62	0.81
Total	36.71 (10.11)	0.95^n.s.^	0.43	0.88	1	0.55	0.97

s.e., skewness error; k.e., kurtosis error; *r*_i.t._, corrected item-total correlation; *d*_i_, difficulty index; α_−*i*_, alpha if item is removed.

1 *P* < 0.05; n.s. = not significant.

### Exploratory factor analysis in first subsample of adolescents (*n*_1_ = 298)

The suitability criteria show that the exploratory factor analysis is appropriate (Kaiser–Meyer–Olkin statistic KMO = 0.97; Bartlett χ^2^ = 1.427; *P* < 0.001; determinant 0.05). The exploratory results gave three factors, each with six items, all factor loadings were above 0.30, no complex items were observed and the inspection of residuals (root mean square residuals) was 0.0443 (below Kelly's criterion) (Table 3). All items were kept for subsequent analysis.

**Table 3:** Rotated factorial matrix of the exploratory factor analysis^a^

	Dimensions			Size of communalities, *h*^2^
SRSA-18 item	Factor 1	Factor 2	Factor 3	
1	**0.70**	−0.02	0.11	0.62
2	**0**.**62**	0.15	−0.28	0.86
5	**0**.**84**	0.24	0.08	0.74
8	**0**.**79**	0.11	−0.17	0.69
17	**0**.**82**	0.04	0.09	0.91
18	**0**.**91**	0.12	−0.02	0.82
4	0.02	**0**.**81**	−0.11	0.84
6	0.11	**0**.**79**	0.02	0.63
9	0.01	**0**.**92**	0.19	0.64
10	−0.23	**0**.**84**	0.22	0.95
11	0.13	**0**.**78**	0.31	0.75
16	0.26	**0**.**82**	0.09	0.71
3	0.16	0.14	**0**.**89**	0.83
7	0.05	0.21	**0**.**84**	0.68
12	−0.04	−0.23	**0**.**81**	0.59
13	0.19	14	**0**.**96**	0.79
14	0.11	0.02	**0**.**92**	0.73
15	0.07	−0.11	**0**.**74**	0.84
% of variance explained	68.22	57.51	42.12	

Factor 1, internal protection; factor 2, emotional stability; factor 3, external protection.

a. Rotated load with values >0.30 are shown in bold.

### Confirmatory factor analysis in second subsample (*n*_2_ = 330)

This sample of adolescents exhibited multivariate normality (Mardia statistic 102.14) and the CFA gave significant values that were less than 3 (χ^2^/d.f. = 2.5 *P* < 0.01: χ^2^ = 139.12; d.f. = 56). Additionally, the root mean square residual (RMR) demonstrated an acceptable fit with values below 0.06 and the goodness-of-fit indices were adequate (RMSEA = 0.02; 95% CI 0.01–0.03; CFI = 0.97; TLI = 0.98; GFI = 0.96).

### Measurement invariance in second subsample (*n*_2_ = 330)

The results for the CFA models specified for boys and girls and for each age group showed a good fit to the data (Table 4). The test of configural invariance (in the baseline model, factor loadings and variances were freely estimated for boys and girls and for each age group), metric invariance (factor loadings were constrained to be equal across gender groups and age groups) and scalar invariances (all item intercepts were forced equal for all items) also exhibited good fit. With respect to gender, the non-statistically significant increase in χ^2^ from the baseline model to the total metric invariance model was 1.27 (Δχ^2^ = 1.27) and the CFI was 0.001, which is below the criterion of 0.01,^33,34^ indicating total metric equivalence between boys and girls. The metric invariance also indicated that there was no significant variation in expectancy between the age groups considered (Δχ^2^ = 4.11); furthermore, ΔCFI was below the criterion (ΔCFI = 0.003). There were no statistically significant differences between the comparison of the baseline model with the scalar model (Δχ^2^ = 3.07) and the CFI increase of 0.002 was well below the recommended maximum. These results lead us to assume that the same construct is measured in the different gender and age groups and that the scale items measure the same variables (i.e. are invariant) in these groups.

**Table 4:** Indices of fit for the invariance tests by gender and age for second subsample (*n*_2_ = 330)

	χ^2^	d.f.	χ^2^/d.f.	*P*	RMSEA (95% CI)	CFI	Δχ^2^	ΔCFI
Boys (*n* = 156)	38.22	21	1.82	0.05	0.02 (0.01–0.03)	0.97		
Girls (*n* = 174)	49.53	21	2.35	0.00	0.03 (0.02–0.04)	0.96		
Configural invariance for gender	86.11	35	2.46	0.28	0.03 (0.02–0.03)	0.98		
Metric invariance for gender	109.12	48	2.27	0.39	0.02 (0.01–0.03)	0.97	1.27^n.s.^	0.001
Scalar invariance for gender	110.07	52	2.12	0.18	0.03 (0.02–0.04)	0.96	1.44^n.s.^	0.002
Age 13–14 years	122.10	52	2.35	0.00	0.01 (0.01–0.02)	0.96		
Age 15–16 years	134.37	54	2.49	0.01	0.03 (0.02–0.04)	0.97		
Age 17–18 years	149.12	66	2.26	0.02	0.03 (0.01–0.03)	0.97		
Configural invariance for age	179.15	73	2.45	0.78	0.01 (0.01–0.02)	0.98		
Metric invariance for age	181.17	93	1.95	0.93	02 (0.02–0.03)	0.95	4.11^n.s.^	0.003
Scalar invariance for age	193.82	96	2.02	0.86	04 (0.03–0.04)	0.98	3.07^n.s.^	0.002

χ^2^/d.f., chi-squared goodness-of-fit index; RMSEA, root mean square error of approximation; CFI, comparative fit index; Δχ^2^, difference test between the configural and metric or scalar invariance models; ΔCFI, difference test between comparative fit index.

*P < 0.05; **P < 0.01; n.s., not significant.

### Reliability and convergent and divergent validity

Internal consistency (ω = 0.91) yielded high values, including for each subdimension: internal protection (i.p.) (ω = 0.88), emotional stability (e.s.) (ω = 0.79) and external protection (e.p.) (ω = 0.82). Convergent validity with the RS-14 total score was high and significant (*r* = 0.93; *P* < 0.01) for the overall score and for the subdimensions (*r*_i.p._ = 0.91; *P* < 0.01; *r*_e.s._ = 0.89; *P* < 0.01; *r*_e.p._ = 0.84; *P* < 0.01). Overall divergent validity with SENTIA was adequate (*r*_SENTIA_ = −0.86; *P* < 0.01), although lower than with RS-14, except for the subdimensions of internal protection (*r*_i.p._ = −0.84; *P* < 0.01) and emotional stability (*r*_e.s._ = −0.82; *P* < 0.01). The relationship with external protection was adequate (*r*_e.p._ = −0.74; *P* < 0.01).

## Discussion

The aim of this study was to adapt the Scale of Resilience to Suicide Attempts (SRSA-18) for use with adolescents, providing data to verify its structure (EFA and CFA), its reliability, and convergent and divergent validity, as well as assessing its invariance by gender and age.

Suicide-related behaviours in adolescence are often unseen and there is a high level of associated stigma, which reduces early detection in the school setting compared with health centres or emergency departments, as other studies have suggested.^36^ However, the use of measures focusing on protective factors, such as the SRSA-18, in adolescents can help in this early detection process. Furthermore, the SRSA-18 does not use words or phrases related to suicide in any of its items. This aspect is crucial at these developmental stages and indicates that the SRSA-18 measures suicide attempts indirectly, focusing more on protective factors than on risk factors, and defining resilience as an outcome, as other studies on resilience have proposed.^37,38^

Factor analyses confirmed a three-dimensional structure for the SRSA-18 scale (internal and external protection and emotional stability). Internal consistency was high in the total score and in each of the subdimensions, and the convergent validity with other resilience scales (RS-14) and divergent validity with a suicide planning and attempts scale (SENTIA) make it especially useful in school and clinical contexts for the prevention of possible suicide attempts. Maintaining the three-dimensional structure is important because this scale is based on a protective factor approach, where the focus is on patterns of adolescent functioning that lead to positive outcomes despite the adverse experience.^8,39^

### Limitations and future research

This study has some limitations. First, the adaptation to a specific country and culture makes it difficult to generalise the results to other countries. However, it also opens up a line of research necessary to test the similarities and differences in the psychometric properties of the SRSA-18 in adolescents from other language groups and cultures. Second, the adaptation process in adolescents entailed some grammatical modifications to the items in the original version of the scale (adapted for adults who have made suicide attempts). These variations may be explained by differences in the way adolescents and adults interact with their social environment and by aspects related to cognitive development at this stage of human development. Finally, it would be interesting to follow up the adolescents to examine the stability of the SRSA-18 measure to help clarify the significance of potential changes in scores.^40^ All of these aspects could be considered as recommended actions for future researchers wishing to further explore this new line of work in adolescents related to resilience based on protective factors.

## Data Availability

The data that support the findings of this study are available from the corresponding author on reasonable request.

## Appendix 1

### Modifications to the SRSA-18 following input from the adolescents in the second pilot sample^a^

**Table:**

Original item	Modified item
1. I always see the glass half full, instead of half empty	1. I see the glass as half-full, rather than half-empty
3. If I have a problem, I ask my family or friends for help	3. If I have a problem, I ask for help from a parent or family member
11. I often think before I act	11. I think before I act
12. There are people who are interested in me and what happens to me	12. There are people who are interested in my life
13. I am able to share my problems with family or friends	13. I talk about my problems to family or friends
14. I have a group of friends to have fun	14. I have a group of friends and I can have fun with them
15. When something worries me I have people who comfort me, listen and encourage me	15. When I am worried about something I have people who comfort me, listen to me and encourage me
17. I know how to get the funny part out of problems	17. I am able to make fun of problems

## Appendix 2

### The SRSA-18 for adolescents^a^

We would like to know about some aspects of your life. Please answer all the questions by placing an (x) on the number as you feel it best applies to your current situation.

**Table:**

	Never	Sometimes	Half of the time	Almost always	Always
1. I see the glass as half-full, rather than half-empty	0	1	2	3	4
2. I am a valuable person	0	1	2	3	4
3. If I have a problem, I ask for help from a parent or family member	0	1	2	3	4
4. I have plans for the future	0	1	2	3	4
5. I take problems with humour	0	1	2	3	4
6. My actions are guided by my head, not my heart	0	1	2	3	4
7. I make friends easily	0	1	2	3	4
8. I am as good at what I do as my peers or friends	0	1	2	3	4
9. I hope to have a happy life	0	1	2	3	4
10. I am able to control my anger	0	1	2	3	4
11. I think before I act	0	1	2	3	4
12. There are people who are interested in my life	0	1	2	3	4
13. I talk about my problems to family or friends	0	1	2	3	4
14. I have a group of friends and I can have fun with them	0	1	2	3	4
15. When I am worried about something I have people who comfort me, listen to me and encourage me	0	1	2	3	4
16. I control my impulses	0	1	2	3	4
17. I am able to make fun of problems	0	1	2	3	4
18. In difficult times, I tend to hope for the best	0	1	2	3	4
